# The role of mitochondrial dysfunction in the cytotoxic synergistic effect of gemcitabine and arsenic on breast cancer

**DOI:** 10.1371/journal.pone.0312424

**Published:** 2025-01-07

**Authors:** Farshid Maleki, Somayeh Handali, Mohsen Rezaei

**Affiliations:** 1 Faculty of Medical Sciences, Department of Toxicology, Tarbiat Modares University, Tehran, Iran; 2 Medical Biomaterials Research Center (MBRC), Tehran University of Medical Sciences, Tehran, Iran; 3 Institute for Natural Products and Medicinal Plants (INPMP), Tarbiat Modares University, Tehran, Iran; China Medical University (Taiwan), TAIWAN

## Abstract

Breast cancer is the most common type of cancer in women worldwide. A common approach to cancer treatment in clinical practice is to use a combination of drugs to enhance the anticancer activity of drugs while reducing their side effects. In this regard, we evaluated the effectiveness of combined treatment with gemcitabine (GCB) and arsenic (ATO) and how they affect the cell death pathway in cancer cells. Cytotoxic activity of drugs individually or combined against MDA-MB-231 and MCF-7 was performed by MTT method and isobolographic analysis was used to determine the interaction between these factors. The combination of ATO and GCB showed synergistic anti-cancer activity (CI < 1) in both cancer cell lines. The combination of ATO and GCB induced sub-G1 phase arrest, apoptosis and death rates in MCF-7 and MDA-MB-231 cells. The apoptotic response induced by the combination of GCB and ATO was dependent on caspase 3/7. Combined treatment with mitochondrial membrane potential (MMP) reduction and increased reactive oxygen species (ROS) production caused mitochondrial dysfunction. Co-treatment significantly reduced catalase (CAT) activity in both cancer cells compared to the control group and cells treated with each monotherapy. A significant decrease in cellular GSH was observed in cancer cells treated with ATO and GCB. In addition, migration and invasion were significantly reduced in breast cancer cells treated with the combination of ATO and GCB compared to cells treated with ATO and GCB. In conclusion, the combined treatment of ATO and GCB synergistically increased the anti-cancer activity, and these findings provide an effective approach for the treatment of breast cancer. To the best of our knowledge, this is the first study showing promising results for combination therapy with ATO and GCB in breast cancer.

## Introduction

Breast cancer is one of the most common cancers in women, which is generally very aggressive with high rate of recurrence [1]. Surgery, chemotherapy, radiotherapy, hormone therapy and immunotherapy are common treatments for breast cancer. Despite significant advances in the treatment of breast cancer, it remains a major threat to women’s health [2]. Combined treatment has been considered significantly in clinics due to the reduction of problems related to chemotherapy drugs [3]. Combinations of drugs are usually more effective since each drug compensates for the other. Moreover, the side effects related to high doses of single drug can be eliminated, because a combination of drugs synergistically improves different biological signaling pathways of drugs and allows the use of low doses of each drug [3,4].

Gemcitabine (GCB), a hydrophilic drug is extensively used in the treatment of different solid tumors such as breast cancer. It is a specific analogue of deoxycytidine that inhibits ribonucleotide reductase, dCMP-deaminase and CTP synthetase and eventually DNA damage [5–8].

Arsenic trioxide (ATO) is an approved anti-cancer agent for the treatment of patients with acute promyelocytic leukemia (APL). Furthermore, it has been reported that ATO shows significant anti-cancer activity on some solid tumors including; gastric, breast, colon, liver, esophageal and lung cancer [9,10]. ATO triggers apoptosis in cancer cells through numerous mechanisms, including induction of oxidative stress, activation of the JNK (c-Jun N-terminal kinase) signaling cascade and suppression of AKT activity [5]. However, higher level of ATO is required for apoptosis induction in cancer cells. Treatment with ATO alone is also restricted in the clinical applications due to its toxicity and low efficacy. Therefore, combining ATO with other anti-cancer drugs may provide a new approach for cancer treatment [5,9].

In the current study, we evaluated whether the combination therapy of ATO and GCB are effective against the breast cancer cell lines. To the best of our knowledge, this is the first study indicated promising results for ATO combined with GCB in the treatment of breast cancer.

## Material and methods

MDA‐MB‐231 (human breast cancer cell) and MCF-7 (human breast cancer) were obtained from Pasteur Institute, Iran. Dulbecco’s modified eagle medium (DMEM), penicillin-streptomycin (Pen-Strep) and phosphate buffered saline (PBS) were purchased from Bioidea, Iran. Fetal bovine serum (FBS) was obtained from Gibco, USA. MTT [3-(4,5-dimethylthiazol-2-yl)-2,5-diphenyltetrazolium bromide], arsenic trioxide (ATO), gemcitabine (GCB), dimethyl sulfoxide (DMSO), propidium iodide (PI), fluorescein diacetate (FDA), dichloro-dihydro-fluorescein diacetate (DCFH-DA), rhodamine 123, sulfosalicylic acid and O-phthalaldehyde (OPT) were purchased from Sigma-Aldrich Co, USA. RNase A and Matrigel were acquired from Bio basic, Canada and Corning Life Sciences, USA, respectively. There were no human or animal studies.

### Cytotoxicity assay

The cytotoxicity of ATO and GCB were determined by MTT assay. MDA‐MB‐231 and MCF-7 cells were grown at 37°C, 5% CO2 and 95% relative humidity in high‐glucose DMEM supplemented with 10% FBS and 1% Pen-Strep. The cells were seeded in a 96-well plate (1×10^4^ cells/well) and allowed to attach and grow for 24 h. Then, cells were treated with different concentrations of ATO (1.25, 2.5, 5, 10 and 20 μM) and GCB (10, 20, 40, 80, 160 and 320 μM) for 48 h. Cells were incubated with MTT (5 mg/ml) at 37˚C for 4 h and 100 μl of DMSO was added to the wells and plates were incubated at 37˚C for 30 min. The cell viability percentages were calculated according the following Eq (1) and IC_50_ was determined by GraphPad Prism version (8.0.1).


Cellviability(%)=ODsample/ODcontrol×100 (1)

### Combination Index (CI) analysis

The isobolographic analysis was performed to determine the interactions between drugs, which allows their stratification as synergistic, additive and antagonistic. For this purpose, the trypan blue exclusion test was used for evaluation the combination effects of ATO and GCB on breast cancer cells. The cells were cultured under the IC_50_ concentration ratio at 0.0625x (5.625:0.625), 0.125x (11.25:1.25), 0.25x (22.5:2.5), 0.5x (45:5), 1x (90:10) μM of GCB and ATO, respectively for 48 h. Then, cells were centrifuged at 1500 rpm for 5 min and cell pellets resuspend in 1 ml serum-free complete medium. 1-part cell suspension and 1-part of 0.4% trypan blue were mixed. The unstained (viable) and stained (nonviable) cells separately were counted by the hemocytometer. The viability percentage of cells were calculated as follows (Eq 2):

Viablecells(%)=(totalnumberofviablecellsperml/totalnumberofcellsperml)*100 (2)

Using this analysis method, the combination index (CI) value was calculated according to the levels of growth inhibition by each agent individually and combination of GCB with ATO. The CI values of <1, >1 and = 1 indicate synergism, antagonism and additive effect of drugs, respectively.

### Apoptosis and cell cycle analysis

MDA‐MB‐231 and MCF-7 cells (5 × 10^5^ cells/well) were seeded in to 6‐well plate and treated with GCB, ATO and GCB+ATO for 48 h. Annexin V-FITC and propidium iodide (PI) were added to each well according to the manufacturer protocol of IQ Products (IQP-116F, Netherlands) kit. Flow cytometry was performed using BD FACSCanto^TM^ II (BD Biosciences, San Jose, CA, USA). For cell cycle analysis, MDA‐MB‐231 and MCF-7 cells (5 × 10^5^ cells/well) were seeded in to 6‐well plate and treated with GCB, ATO and GCB+ATO for 48 h. Then cells were suspended in 70% cold ethanol and incubated in 4°C for 4 h. After centrifugation, the cell pellets were resuspended with PI (50 μg/ml), RNase A (10 μg/ml) and PBS (940 μl) and incubated in the dark for 30 min in 37°C. Cell cycle distributions were also determined using BD FACSCanto^TM^ II.

### Fluorescein Diacetate (FDA) and Propidium Iodide (PI) double staining

MDA‐MB‐231 and MCF-7 cells (5 × 10^5^ cells/well) were seeded in to 6‐well plate and treated with GCB, ATO and GCB+ATO for 48 h. Then, cells were washed with PBS and stained with FDA (10 μg/ml) and PI (50 μg/ml) on ice for 5 min. After that, FDA-PI working solution was aspirated, then cold PBS was added and images were taken by fluorescent microscopy (Olympus IX71, Japan).

### Caspase 3/7 activity assay

Caspase-3/7 activity was determined using the kit of Kiazist (KCAS96, Iran) according to the manufacturer’s protocol. 1 × 10^6^ cells of MDA‐MB‐231 and MCF-7 were treated with GCB, ATO and GCB+ATO for 48 h. Cells were harvested and incubated with 500 μl of caspase lysis buffer for 20 min at 4°C. Then, cells were centrifuged at 12000 rpm for 15 min at 4°C and supernatants were used to measure caspase 3/7 activity. The assay is based on the formation of the chromophore p-nitroaniline (p-NA) formed by cleavage from the labeled substrate DEVD-pNA. The p-NA can be quantified using a spectrophotometer or a microtiter plate reader reading absorbance at 405 nm.

### Detection of intracellular ROS

Intracellular ROS was detected using DCFH-DA. After treatment cells with GCB, ATO and GCB+ATO for 48 h, cells were incubated with 20 μmol/L DCFH-DA at 37°C for 30 min in the dark. The fluorescence intensity of 2,7-dichlorofluorescein (DCF) as fluorescent compound (that produced due to deacetylated a DCFH-DA by intracellularly esterase) was detected by spectrofluorometer (CYTATION 3, Imaging reader, USA).

### Analysis of mitochondrial membrane potential

Cells were treated with GCB, ATO and GCB+ATO for 48 h. Then, cells were incubated with 20 μM of rhodamine 123 dye for 40 min at 37°C. After incubation, the emission and excitation signals were detected at 535 and 490 nm, respectively using a spectrofluorometer (CYTATION 3, Imaging reader, USA).

### Intracellular GSH and CAT activity assay

The GSH content of cells were measured as previous described [11]. Briefly, 1 × 10^6^ cells of MDA‐MB‐231 and MCF-7 were treated with GCB, ATO and GCB+ATO for 48 h. Then, cells were harvested and sonicated to lyse. Samples were centrifuged at 18000 *g* for 10 min at 4°C and the supernatants collected and mix with 5% of sulfosalicylic acid and centrifuged again at 9000 *g* for 10 min at 4°C. For GSH assessment, O-phthalaldehyde was added to the supernatant and kept at room temperature for 10 min. Then, GSH activity was assessed at excitation of 355 nm and emission of 420 nm using a spectrofluorometer (CYTATION 3, Imaging reader, USA). Catalase (CAT) activity was also determined using the kit of Kiazist (KCAT96, Iran) according to the manufacturer’s protocol.

### Invasion assay

24 transwells cell culture insert with 8 μm pore size were used for invasion assay. At the first, 40 μl Matrigel was added to a 24 transwell insert and incubated in a 37°C for 30 min in order to solidification. MDA‐MB‐231 and MCF-7 cells (3 × 10^4^ cells/well) were seeded into 24‐well plate and treated with GCB, ATO and GCB+ATO for 48 h. Cells were harvested and resuspended in 200 μl serum‐free DMEM placed into the upper chamber of each insert. DMEM with 20% FBS, as chemoattractant was added to the bottom chamber. After 24 h, remaining cells and Matrigel were removed with gently swabbing and cells were fixed with 70% ethanol for 10 min to allow cell fixation. Then, cells were stained with 0.2% crystal violet for 15 min. The crystals violet from the top of the membrane were gently removed and allow the transwell membrane to dry. The numbers of invaded cells were counted under an inverted microscope (Olympus IX71. Japan) and counted the number of cells in different fields of view to got an average sum of cells that have migrated through the membrane.

### Migration assay

For migration assay MDA‐MB‐231 and MCF-7 cells were seeded into 6‐well plate (5 × 10^5^ cells/well) with high‐glucose DMEM supplemented with 1% FBS and incubated overnight. When cells reached 90% confluency, using a 10 μl pipette tip, a straight scratch wound was created in each well. Then, wells were gently washed to remove cell debris. This point was considered the “0 h,” and the “zero wound” was captured using a microscope (Olympus IX71) and cells were treated with GCB, ATO and GCB+ATO for 48 h. After that, images of scratch wounds of control and treated groups were captured in phase contrast. Wound areas were measured using the Image J software (National Institutes of Health, Bethesda, MD, USA). The closure of the scratch was quantified according to the following Eq (3):

%ofwoundclosurerate:[(areaattimezero–areaattime48h)/areaattimezero]×100 (3)

### Statistical analysis

Statistical analysis performed using one-way analysis of variance by GraphPad Prism version (8.0) followed by post hoc Tukey’s test. Results were reported as mean ± SD. Flow jo software version 7.6.1 was also used to analyze flow cytometry data. Statistical significance was indicated as *p < 0.05, **p < 0.01 and ***p < 0.001.

## Results and discussion

### Cytotoxicity of GCB and ATO on breast cancer cell lines

**Fig 1:**
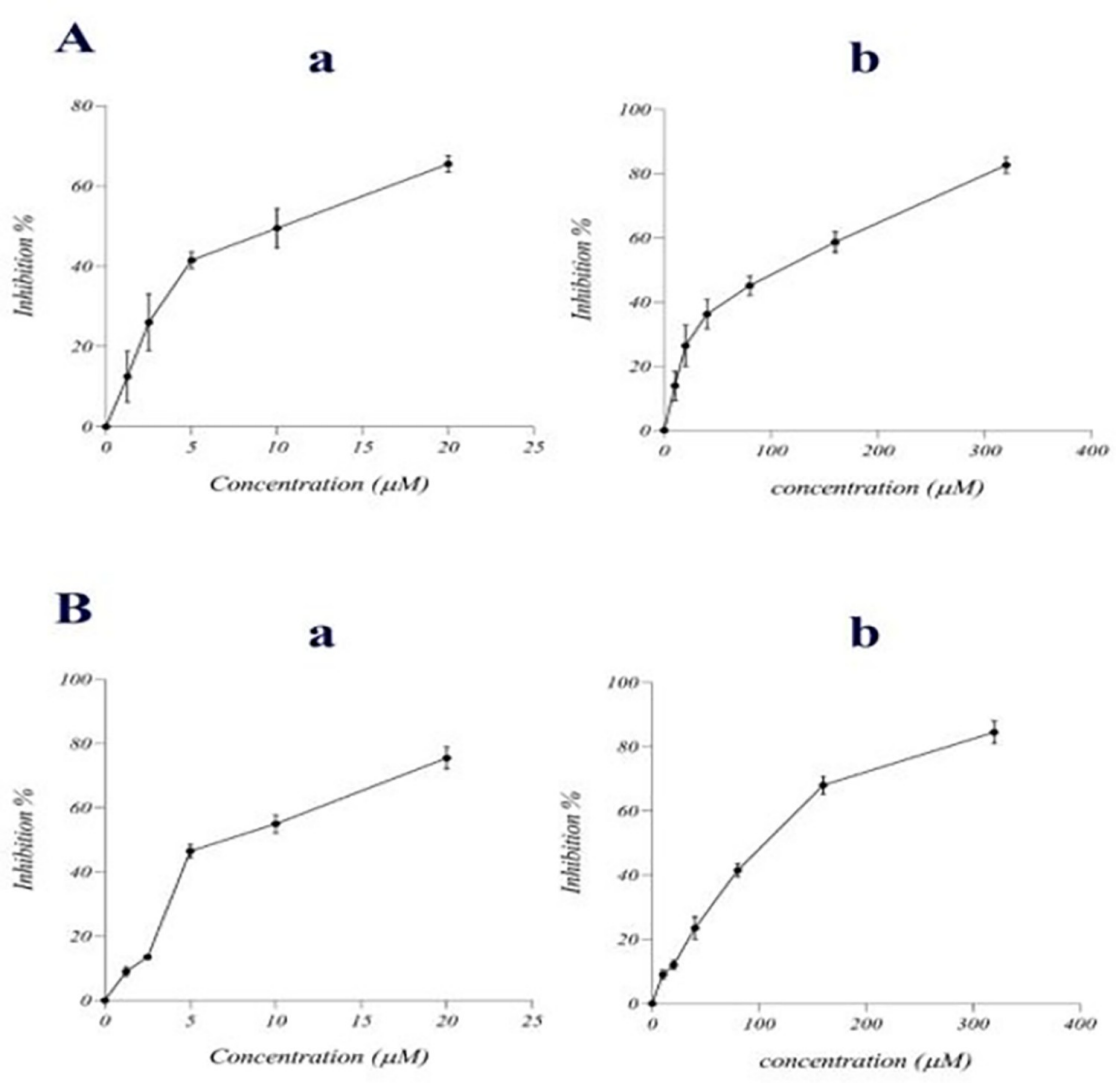
Cytotoxicity activity of (Aa) ATO on MCF-7 cells, (Ab) GCB on MCF-7 cells, (Ba) ATO on MDA-MB-231 cells and (Bb) GCB on MDA-MB-231 cells. Data are presented as mean ± SD (n = 3).

MTT assay was used for cytotoxic evaluation of ATO and GCB on MCF7 and MDA‐MB‐231 cells. After treatment of MCF7 and MDA-MB-231 cells for 48 h with ATO, IC_50_s were obtained at 9.75 ± 2.35 μM and 7.64 ± 1.36 μM, respectively (Fig 1Aa and 1Ba). The IC_50_s of MCF-7 and MDA‐MB‐231 cells treated with GCB were 90.78 ± 14 and 99.64 ± 8.24 μM, respectively (Fig 1Ab and 1Bb). According to the obtained results, cell viability decreased significantly with increasing concentration of drugs. In other words, the cytotoxic effects of ATO and GCB were dose-dependent. Therefore, the combination of 10 μM ATO and 90 μM GCB (diluted concentration till 1:16 ratio) was used for combination treatment. The combination index (CI) was also used for analyzing results. Isobolographic analysis is a statistical method that has been widely used to assess drug interactions. This method is based on the cytotoxicity experiments completed on treated cells with both drugs and the mixture [12]. The ATO and GCB combination showed synergistic anti-cancer activity (CI < 1) in both cancer cell lines (CI: 0.83 for MCF-7 and 0.88 for MDA-MB-231). This finding is supported by data derived *via* trypan blue staining (Table 1), which indicated a decreased viability of MCF-7 and MDA-MB-231 cells treated with both ATO and GCB. Therefore, 4x dilution of IC_50_ (2.5 μM ATO and 22.5 μM GCB) was used for further assessment. According to these findings, the combination of ATO and GCB significantly reduced the IC_50_ of each agent alone. These results showed that by combining ATO and GCB, the side effects of both drugs can be significantly decreased by reducing the dose of both drugs and at the same time maintaining their clinical effectiveness.

**Table 1:** Trypan blue dye exclusion assay for cell viability evaluation (% of control).

**Group**	**1**	**2**	**3**	**4**	**5**
**MCF-7**	94.67 ± 1.53	79.67 ± 2.02	54.90 ± 4.13	22.83 ± 4.04	7.67 ± 2.52
**MDA-MB-231**	84.07 ± 2.96	68.37 ± 2.44	47.58 ± 2.77	18.10 ± 3.03	8.30 ± 3.54

Trypan blue exclusion assay was used to calculate toxic effect of combination ATO and GCB on cells viability after treatment with ATO and GCB (group 1: 0.625 μM of ATO + 5.625 μM of GCB, group 2: 1.25 μM of ATO + 11.25 μM of GCB, group 3: 2.5 μM of ATO + 22.5 μM of GCB, group 4: 5 μM of ATO + 45 μM of GCB and group 5: 10 μM of ATO + 90 μM of GCB) for 48 h. Data are presented as mean ± SD (n = 3).

### Combination effect of ATO and GCB on apoptosis induction

**Fig 2:**
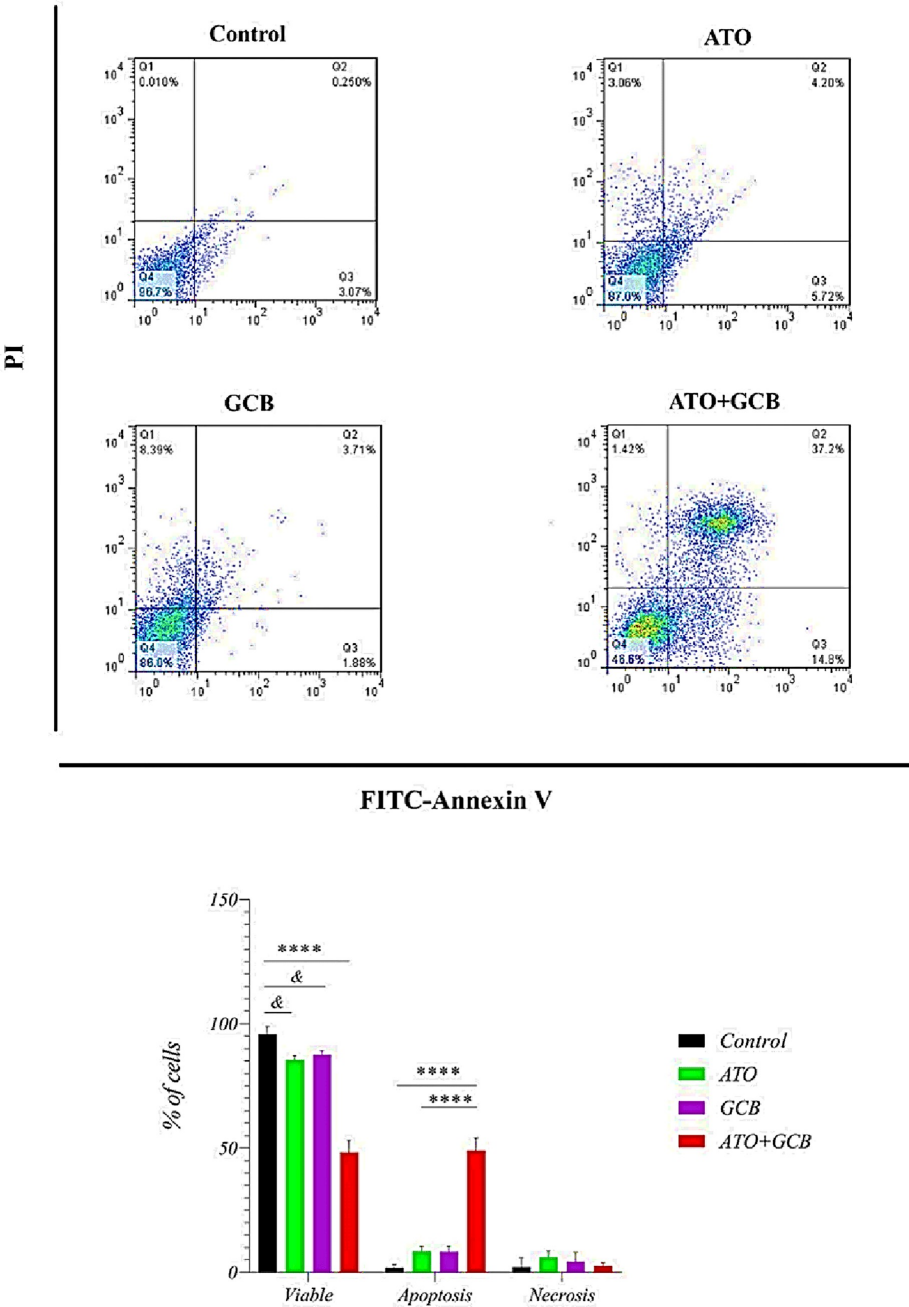
Rate of apoptosis in MCF-7 cells (ATO: 2.5 μM and GCB: 22.5 μM, 48 h, at 37°C). Data are presented as mean ± SD. ****: Significant difference with control, ATO and GCB group (*p* < 0.0001). *&*: Significant difference with control group (*p* < 0.05).

**Fig 3:**
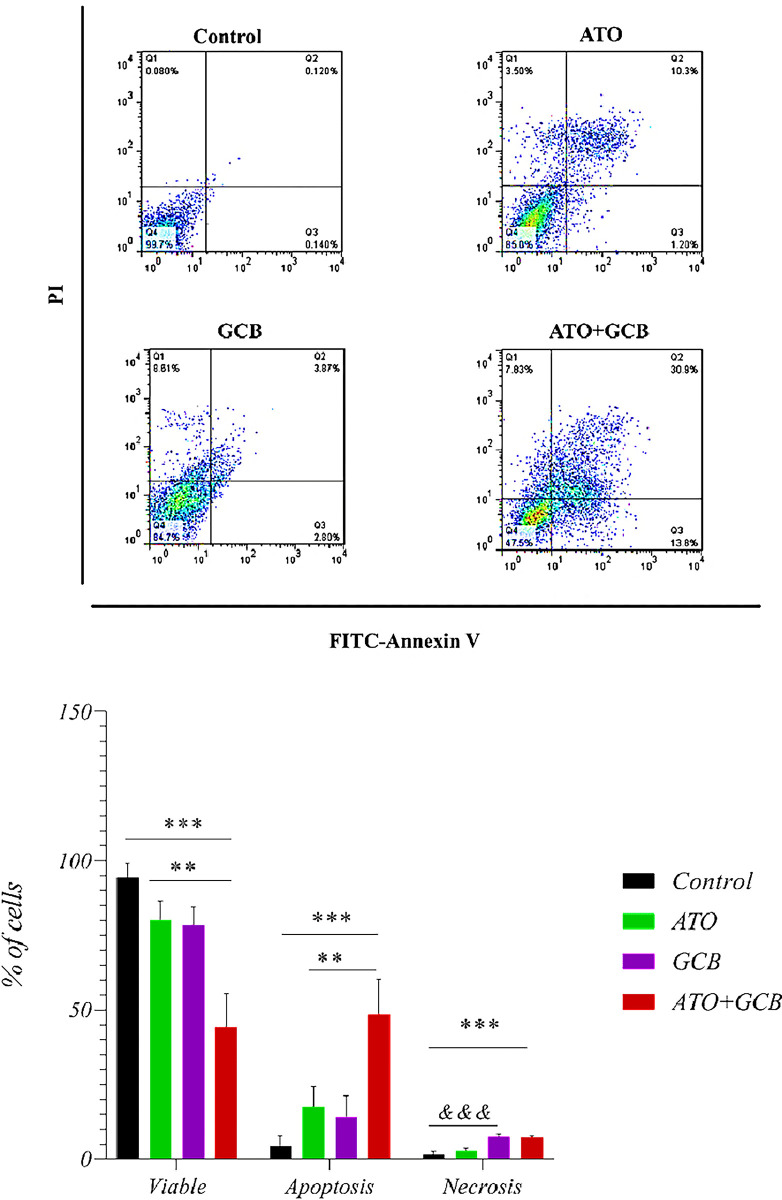
Rate of apoptosis in MDA-MB-231 cells (ATO: 2.5 μM and GCB: 22.5 μM, 48 h, at 37°C). Data are presented as mean ± SD (n = 3). ***: Significant difference with control group (*p* < 0.001). **: Significant difference with ATO, GCB group (*p* < 0.01). &&&: Significant difference with control group (*p* < 0.001).

As shown in Fig 2, combination of ATO and GCB induced higher apoptotic rate in MCF-7 cells as compared to each drugs alone. The rate of apoptosis in MCF-7 cells treated with ATO+GCB was 49.02 ± 5.05% *vs* control 1.88 ± 1.24% (*p* < 0.0001). The rate of apoptosis in MDA‐MB‐231 cells was 48.57 ± 11.69% *vs* control 4.27 ± 3.54%. As shown in Fig 3, ATO+GCB significantly increased apoptosis in MDA‐MB‐231 cells than other groups (control group and groups treated with ATO and GCB alone). These findings showed that combined treatment with ATO and GCB led to a synergistic effect in causing apoptosis in cells. However, it should be noted that treatment with ATO and GCB induced both types of cell death, necrosis and apoptosis, but strongly induced cell death toward apoptosis. Apoptosis is one of the most important pathways of programmed cell death, which plays an important role in cancer prevention [13]. One of the main goals of cancer treatment is to initiate the apoptotic pathway because apoptotic cells are eventually removed by phagocytosis, thus preventing damage to the surrounding tissues. However, the necrosis pathway induces an inflammatory response that leads to damage to adjacent healthy cells.

**Fig 4:**
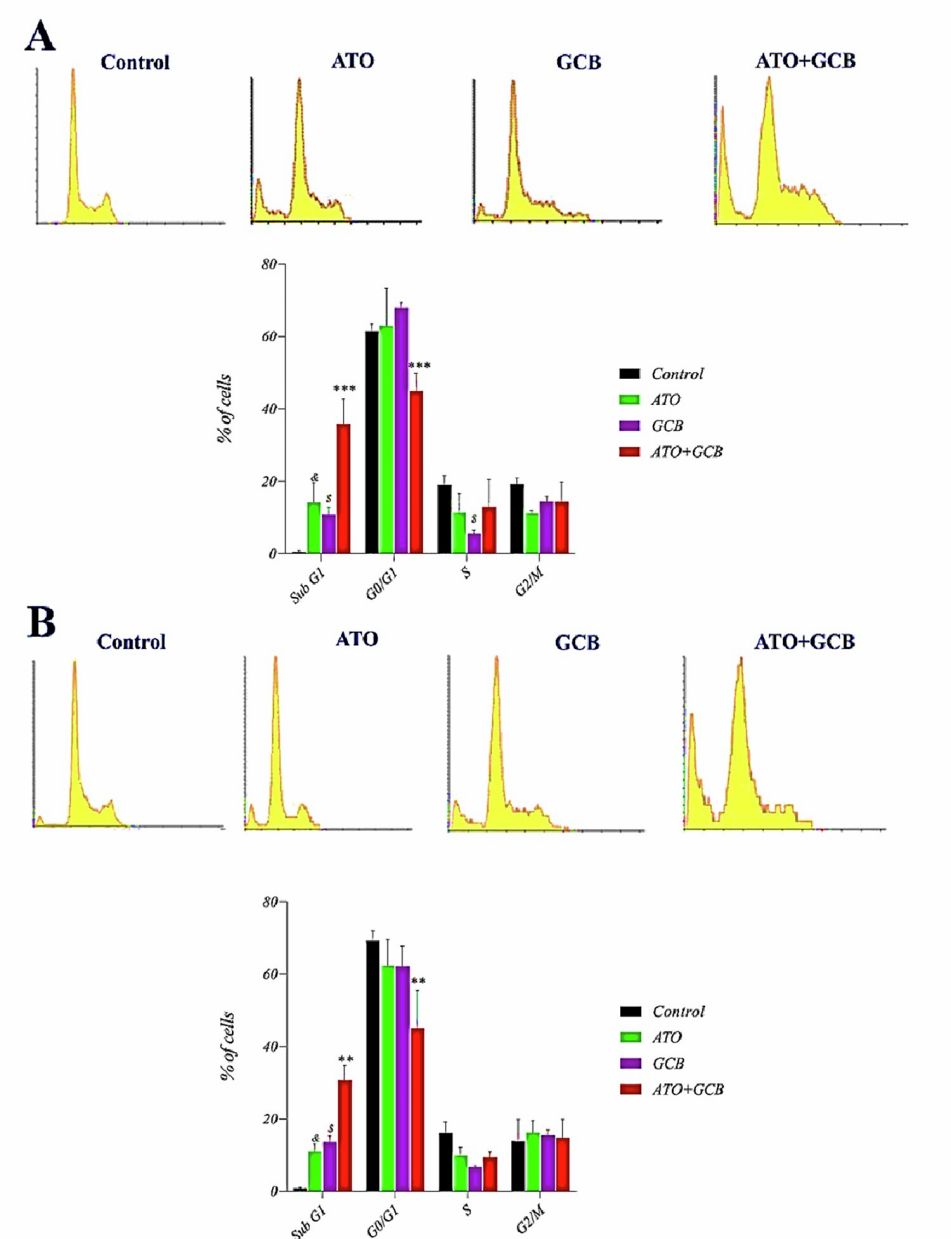
Cell cycle arrest after treatment of A) MCF-7 and B) MDA-MB-231 cells with ATO (2.5 μM) and GCB (22.5) μM for 48 h at 37°C. Data are presented as mean ± SD (n = 3). ***: Significant difference with control group (*p* < 0.001). **: Significant difference with control, ATO, GCB group (*p* < 0.01). *&*: Significant difference with control group (*p* < 0.05). *$*: Significant difference with control group (*p* < 0.01).

We further assayed the effects of ATO+GCB on cell cycle arrest using PI staining on both cell lines. As shown in Fig 4A and 4B, in comparison with control cells, the percentage of cells in sub-G1 phase was clearly increased in MCF-7 cells (ATO+GCB 35.82 ± 6.9% *vs* control 0.43 ± 0.38%), while the percentage of G0/G1 phase cells was noticeably reduced (ATO+GCB 44.9 ± 4.84% *vs* control 61.45 ± 2%). Moreover, the percentage of cells in sub-G1 phase was remarkably increased in MDA‐MB‐231 (ATO+GCB 30.75 ± 4.03% *vs* control 0.85 ± 0.21%), whereas the percentage of cells in G0/G1 phase was decreased (ATO+GCB 45.10 ± 10.35% *vs* control 69.29 ± 2.72%) when compared with control cells or cells treated with either ATO or GCB alone. Cell cycle is crucial for cell growth, which controls cell proliferation [14]. These results demonstrated that combination of ATO and GCB synergistically increased sub-G1 phase arrest in MCF-7 and MDA‐MB‐231 cells, representing the apoptosis induction owing to DNA fragmentation which seems to be the main anti-cancer mechanism of ATO and GCB [15,16].

### FDA-PI staining and viability of cells

**Fig 5:**
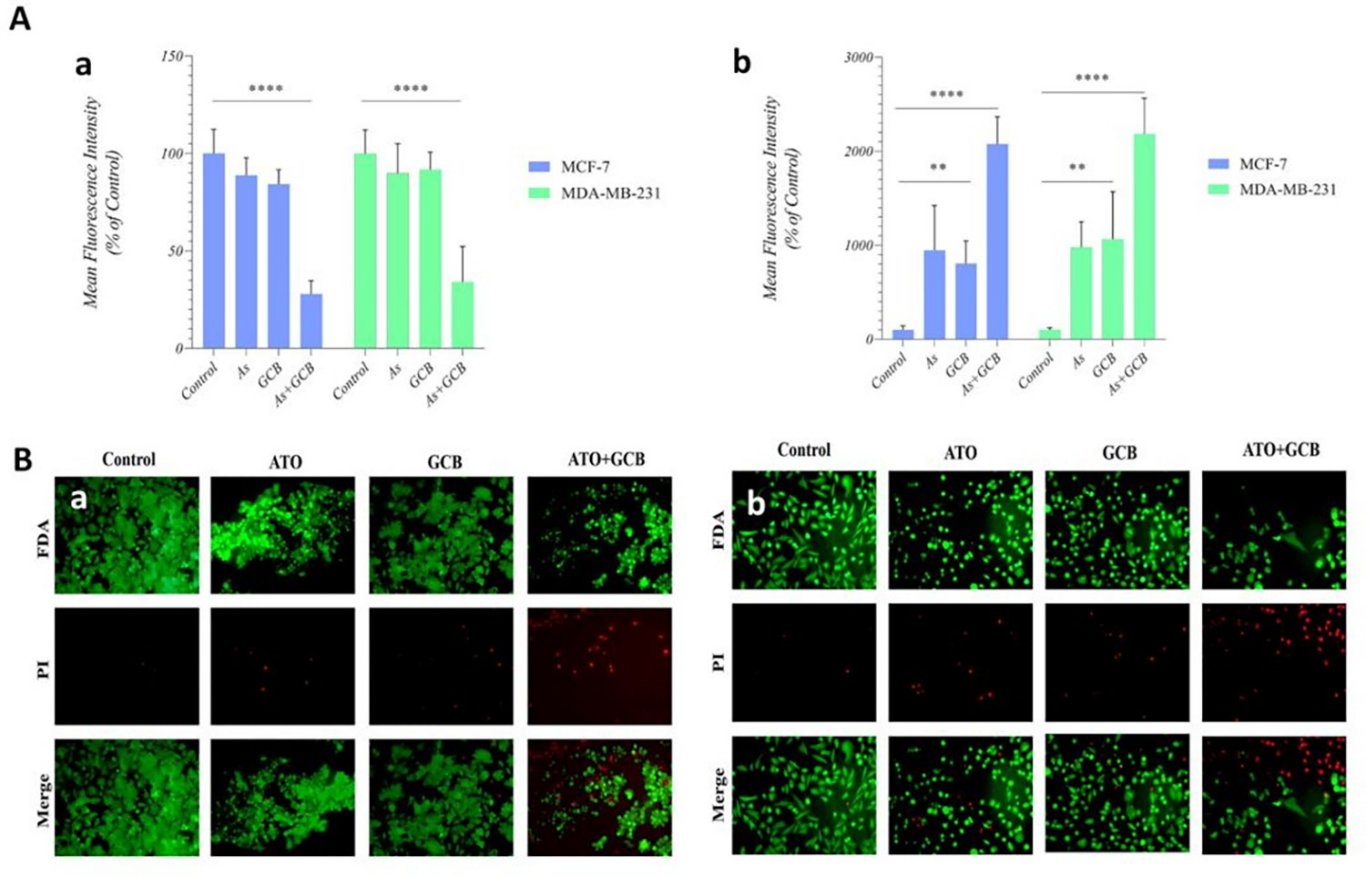
Mean fluorescence intensity of (Aa) FDA, (Ab) PI, cell viability as determined by FDA-PI staining. (Ba) MCF-7 and (Bb) MDA-MB-231 cells stained with FDA/PI (alive cells stained green, whereas dead cells stained red).

In viable cells, fluorescein diacetate (FDA, a nonpolar ester) can easily cross the cell membranes and after being hydrolyzed by free exoenzymes and membrane-bound enzymes, fluorescein is released and remains in the cells with healthy membranes [17]. Propidium Iodide (PI) interacts with DNA only in damaged cells; therefore, it is used for detection of dead cells [18]. In the current study, FDA-PI double staining was used to detect cell viability by evaluating the dead and viable cells. According to the obtained results, combination of ATO+GCB remarkably increased the rate of death in both cancer cells more than cells treated with ATO and GCB alone (Fig 5A and 5B).

### Caspase 3/7 activity assay

**Fig 6:**
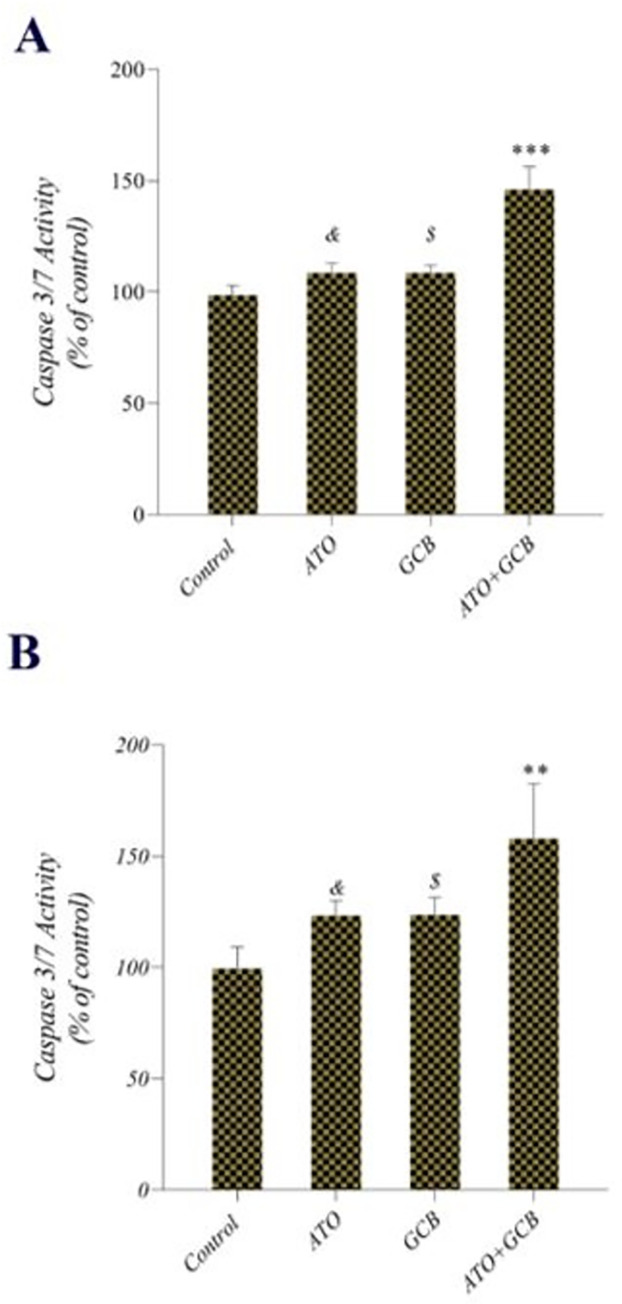
Caspase 3/7 activity after treatment of A) MCF-7 and B) MDA-Mb-231 with ATO (2.5 μM) +GCB (22.5 μM) for 48 h at 37°C (n: 3). Data are presented as mean ± SD. ***: Significant difference with control group (*p* < 0.001). **: Significant difference with control group (*p* < 0.01). *&*: Significant difference with control group (*p* < 0.05). *$*: Significant difference with control group (*p* < 0.05).

To determine whether the induction of apoptosis after treatment with GCB and ATO is dependent on caspase 3/7, cells were treated with the IC_50_ of GCB and ATO. As shown in Fig 6, monotherapy with ATO or GCB significantly increased the activity of caspase 3/7 in MCF-7 and MDA‐MB‐231 cells more than control group. Interestingly, the combined therapy of ATO+GCB remarkably elevated the activity of caspase 3/7 in cancer cells compared to those of cells treated with each monotherapy. Caspases are a unique family of cysteine proteases that trigger apoptosis in cells [19] and caspase 3/7 plays a crucial role in the process of apoptosis [20]. According to obtained results, the apoptotic response induced by GCB and ATO was dependent on caspase 3/7. These findings are consistent with the results of other researchers that reported different combinations of chemotherapeutic agents can significantly induce caspase 3/7 activity in cancer cells [21,22].

### Determination of intracellular ROS and MMP

**Fig 7:**
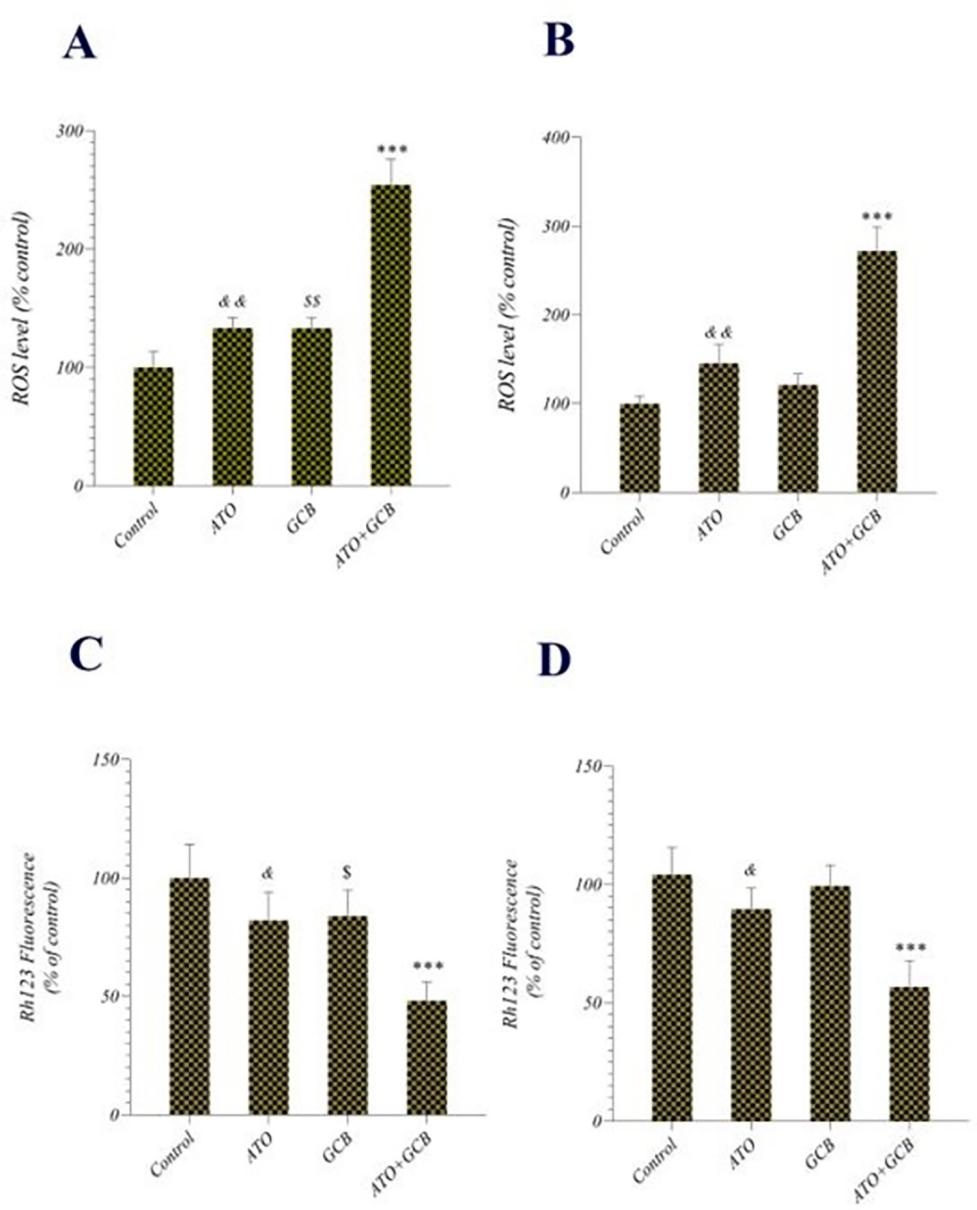
Relative ROS production in A) MCF-7 and B) MDA-Mb-231 cells treated with ATO, GCB and ATO + GCB and measurement of MMP in C) MCF-7 and D) MDA-Mb-231 cells treated with ATO, GCB and ATO + GCB (ATO: 2.5 μM+GCB: 22.5 μM, 48 h at 37°C). Data are presented as mean ± SD. ***: Significant difference with control group (*p* < 0.001). **: Significant difference with control group (*p* < 0.01). *&&*: Significant difference with control group (*p* < 0.01). *&*: Significant difference with control group (*p* < 0.05). *$*: Significant difference with control group (*p* < 0.05). *$ $*: Significant difference with control group (*p* < 0.01).

ROS generation in cells treated with ATO+GCB combination was measured to evaluate the oxidative stress status. As illustrated in Fig 7A and 7B, ATO+GCB combination significantly increased the ROS level in cancer cells compared to both drugs alone. It has been reported that high level of ROS causes disruption of mitochondrial membrane potential (MMP) and leads to mitochondrial dysfunction. Mitochondrial dysfunction triggers apoptosis through the cytochrome c release, which promotes the caspase cascade [23]. Consequently, we determined the levels of MMP in MCF-7 and MDA-Mb-231 cells treated with ATO+GCB. It was found that the levels of MMP in co-treatment groups was significantly lower than other groups (*p* < 0.001) (Fig 7C and 7D). These results indicated that combination treatment triggers mitochondrial dysfunction through reduced levels of MMP owing to increased ROS production. Our results are in accordance with Wu et al. findings which observed that co-treatment of ATO and paclitaxel reduced MMP and increased ROS generation [24] and also with the results of Lee et al. which found that combined therapy of GCB and Ivermectin induced apoptotic pathway in cancer cells *via* the overproduction of ROS and disrupting MMP [25].

### Intracellular CAT and GSH activity assay

**Fig 8:**
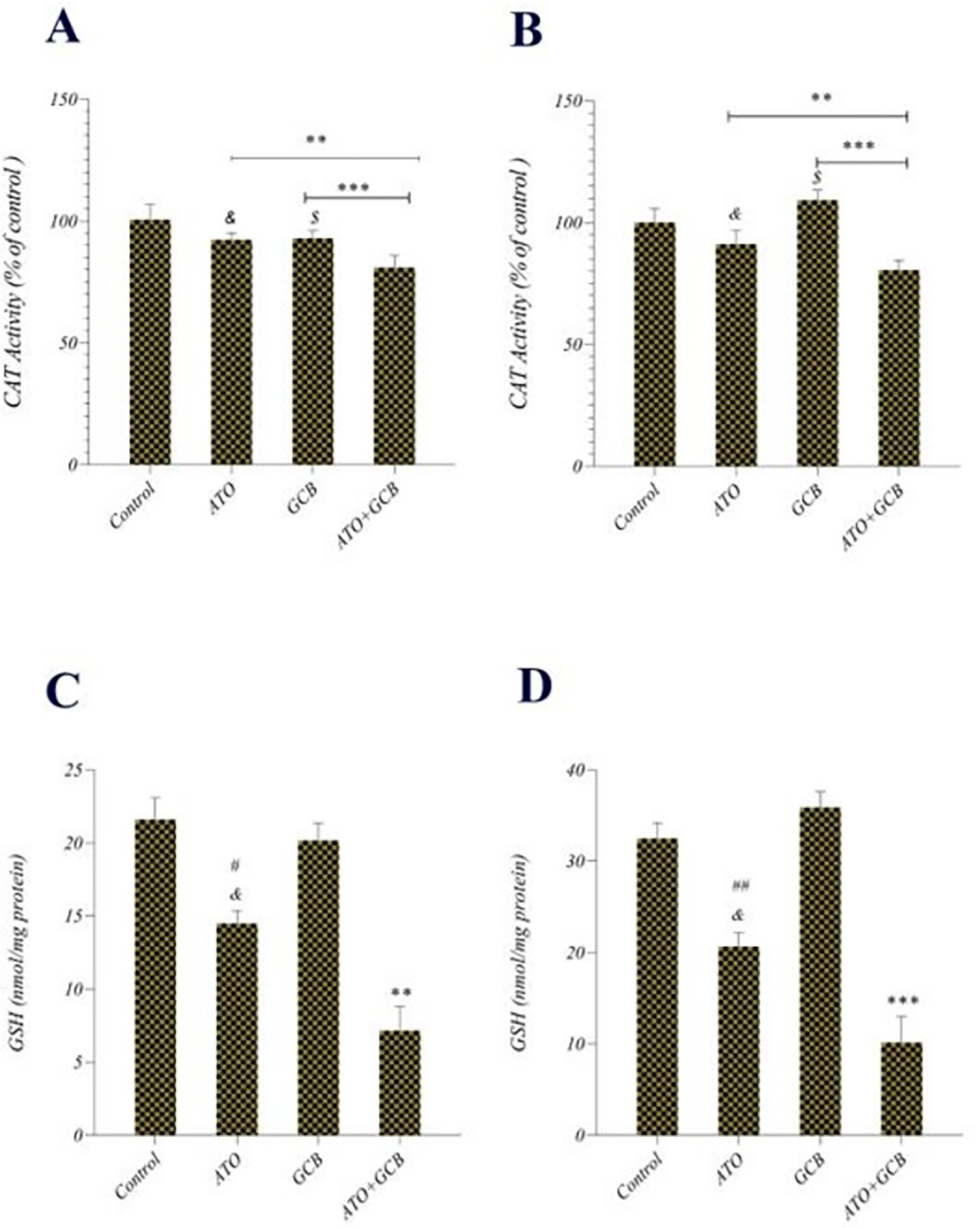
The CAT activity in A) MCF-7 and B) MDA-Mb-231 cells treated with ATO, GCB and ATO + GCB and GSH content in C) MCF-7 and D) MDA-Mb-231 cells treated with ATO, GCB and ATO + GCB (ATO: 2.5 μM+GCB: 22.5 μM), for 48 h at 37°C. Data are presented as mean ± SD. ***: Significant difference with control and GCB group (*p* < 0.001). **: Significant difference with control, ATO, GCB group (*p* < 0.01). *&*: Significant difference with control group (*p* < 0.05). *#*: Significant difference between GCB and ATO group (*p* < 0.05). *##*: Significant difference between GCB and ATO group (*p* < 0.01). *$*: Significant difference with control group (*p* < 0.05). *$ $*: Significant difference with control group (*p* < 0.01).

Catalase decomposes hydrogen peroxide (H_2_O_2_) into water and oxygen, which protects cells from the toxic effects of ROS [26]. ATO+GCB combination caused a statistically significant decrease in the CAT activity in both cancer cells than the control group and cells treated with each monotherapy (Fig 8A and 8B). It has been observed that ATO reduced catalase expression in breast cancer cells [27,28]. Increased levels of catalase have been reported to decrease the effective levels of ROS; consequently, leads to resistance to GCB in cancer cells [29]. According to the results of this study, it seems that ATO decreases catalase activity in breast cancer cells and sensitizes them to GCB.

A significant depletion in cellular GSH was observed in cancer cells treated with ATO+GCB (Fig 8C and 8D). GSH is the most abundant cellular antioxidant that plays an important role in promoting cell survival. Moreover, increasing intracellular GSH is related to resistance of cells to anti-cancer drugs [30,31]. Thus, reducing intracellular GSH enhances ROS production and increases chemotherapy sensitivity. As shown in Fig 8C and 8D, GCB treatment induced GSH synthesis in cancer cells, which was declined by co-treatment with ATO. Previous studies demonstrated that GCB induces GSH synthesis *via* activating of the nuclear factor erythroid 2-related factor 2 (NRF2) antioxidant pathway to counteract the effects of ROS [32]. On the other hand, ATO can reduce GSH synthesis by decreasing intracellular cysteine and glutamate levels [33]. These findings support our hypothesis that ATO might improve the efficacy of GCB on cancer cells by inhibition of GSH synthesis.

### Effect of ATO and GCB combination on the invasion and migration of cancer cells

**Fig 9:**
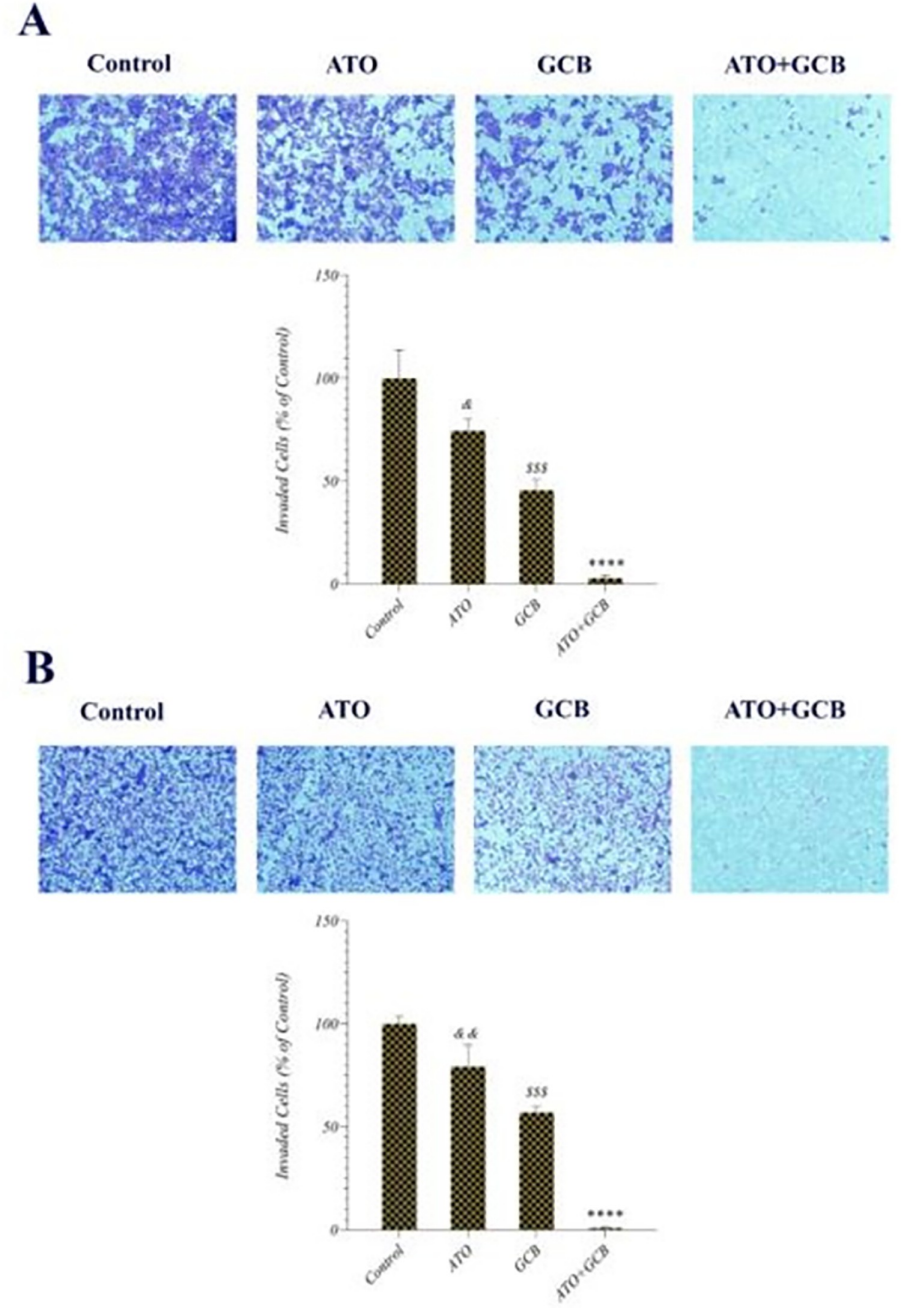
Inhibition of the invasion of A) MCF-7 and B) MDA-Mb-231 cells after treatment with ATO (2.5 μM) and GCB (22.5 μM) for 48 h at 37°C. Data are presented as mean ± SD. ****: Significant difference with control, ATO, GCB group (*p* < 0.0001). *$ $ $*: Significant difference with control group (*p* < 0.001). *&*: Significant difference with control group (*p* < 0.05). *&&*: Significant difference with control group (*p* < 0.01).

**Fig 10:**
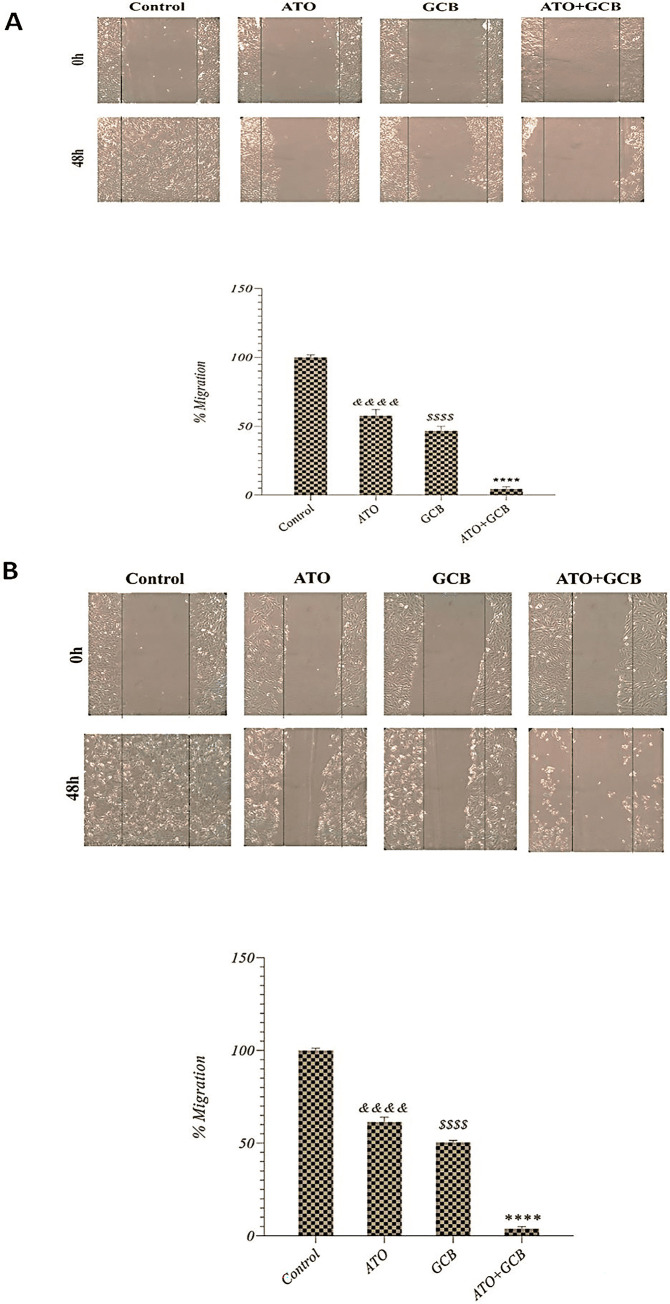
Inhibition of the migration of A) MCF-7 and B) MDA-Mb-231 cells after treatment with ATO (2.5 μM) and GCB (22.5 μM) for 48 h at 37°C. Data are presented as mean ± SD. ****: Significant difference with control, ATO, GCB group (*p* < 0.0001). *$ $ $ $*: Significant difference with control group (*p* < 0.0001). *&&&&*: Significant difference with control group (*p* < 0.0001).

Migration and invasion ability of cancer cells allow them to alter their position in tissues and leads to metastasis, which is the main cause of death in cancer patients [34]. Accordingly, in this study, we evaluated whether the combination of ATO and GCB can improve the ability of both drugs to inhibit breast cancer cells invasion and migration. It was observed that the combination treatment with ATO and GCB could remarkably reduce the rate of invasion in MCF-7 and MDA‐MB‐231 cells (Fig 9A and 9B). Notch signaling pathway plays an oncogenic role in breast cancer progression. It has been reported that ATO inhibits the Notch pathway by down-regulating the expression of Bcl-2 and NF-κB, which leads to inhibition of invasion [35,36]. It was also observed that migration was significantly reduced in MCF-7 and MDA‐MB‐231 cells treated with ATO+GCB combination as compared to those treated with ATO or GCB (Fig 10A and 10B). These results indicate that the combination of ATO and GCB significantly enhances the inhibitory effect on migration and invasion of breast cancer cells. These findings are in agreement with Lin et al. results that reported co-treatment of ATO and berberine increased the inhibition of migration and invasion on cancer cells [37]. In addition, this is the first report of a strategy involving the combination of ATO and GCB in the treatment of breast cancer cells to reduce migration and invasion, which leads to inhibition of metastasis and increases the efficacy of treatment.

Breast cancer is one of the main causes of death in women worldwide, which has become a serious threat to women’s health. GCB is widely used in the treatment of different solid tumors such as breast cancer. ATO also displays significant anti-cancer activity on some solid tumors including breast cancer. However, treatment with GCB or ATO alone is restricted in the clinical applications due to their toxicity and low efficacy. In order to reduce the problems related to chemotherapy drugs, combination therapy has been significantly considered in clinics. It is reported that ATO and GCB reduced cell viability in a manner that was both dose- and time-dependent and also triggering cell cycle arrest and apoptosis in Lymphoma cell lines [38]. The S-phase kinase associated protein 2 (Skp2), which belongs to the F-box protein family, plays a key role in regulating cell cycle progression and is notably overexpressed in pancreatic cancer (PC). In a study, it was demonstrated that ATO inhibited cell growth and invasion by downregulating Skp2 in PC cells. Emerging evidence indicates that Skp2 is critical in mediating drug resistance across various cancer types. Also, it was found that ATO could enhance the sensitivity of PC cell lines to GCB. The results showed that the combination of ATO and GCB produced significant antitumor effects in Patu8988 and Panc-1 PC cells. Furthermore, ATO enhanced the efficacy of GCB through the downregulation of the Skp2 pathway in these cells [39].

**Fig 11:**
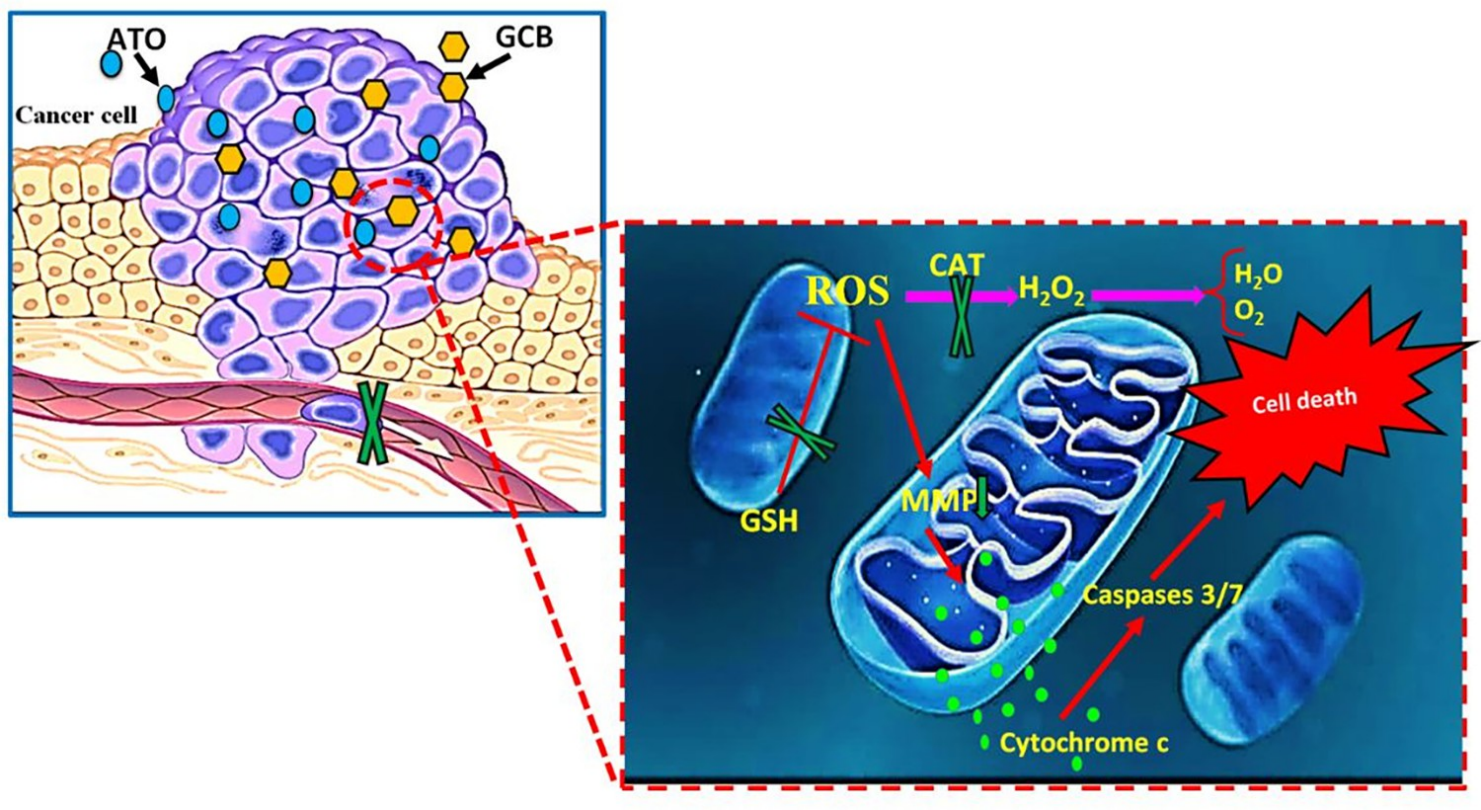
Schematic presentation of mechanism of anti-cancer activity of ATO and GCB combination in breast cancer cells.

In the current study, isobologram analysis was performed to evaluate whether the biological responses resulting from a mixture of agents are superior, less or equal than what would be expected based on the individual activities of each agent alone. According to the obtained results, combination of ATO and GCB showed synergistic anti-cancer activity (CI < 1) in breast cancer cells. Co-administration of ATO and GCB triggered apoptosis *via* the mitochondrial pathway due to increasing the level of ROS, led to disruption of MMP which promoted the caspase cascade in cancer cells. Combination of ATO and GCB significantly improved the inhibitory effect on migration and invasion of breast cancer cells that can be more effective in inhibiting metastatic progression in patients. In addition, the main finding has been that the combination of ATO and GCB has the ability to reduce the dose of each drug alone, their effectiveness is also maintained, and as a result, side effects will be less. In conclusion, the combined treatment of ATO and GCB may be a promising therapeutic strategy to increase the survival rate of breast cancer patients. The proposed mechanism of anti-tumor activity for synergistic effect of ATO and GCB in breast cancer cells is presented schematically in Fig 11.

## Conclusion

The aim of this study was to investigate whether the combination of GCB and ATO shows a stronger therapeutic effect than these agents alone in breast cancer. GCB with ATO showed a synergistic effect on anti-tumor response in cancer cells. According to the findings, it is suggested that the synergistic anti-cancer effect of GCB and ATO on breast cancer cells is done through apoptosis due to increased ROS production, decreased MMP and increased caspase activity. In addition, a significant decrease in cellular GSH, migration and invasion were observed in cancer cells treated with the combination of ATO and GCB. To the best of our knowledge, this is the first report to determine the synergistic effect of ATO and GCB in breast cancer. On the other hand, due to the need for a low dose of GCB and ATO and thus reducing side effects, this combination therapy can be a promising platform for the treatment of breast cancer.
